# Representational changes of latent strategies in rat medial prefrontal cortex precede changes in behaviour

**DOI:** 10.1038/ncomms12830

**Published:** 2016-09-22

**Authors:** Nathaniel James Powell, A. David Redish

**Affiliations:** 1 Graduate Program in Neuroscience, University of Minnesota, Minneapolis, Minnesota 55455, USA; 2 Department of Neuroscience, University of Minnesota, Minneapolis, Minnesota 55455, USA

## Abstract

The ability to change behavioural strategies in the face of a changing world has been linked to the integrity of medial prefrontal cortex (mPFC) function in several species. While recording studies have found that mPFC representations reflect the strategy being used, lesion studies suggest that mPFC is necessary for changing strategy. Here we examine the relationship between representational changes in mPFC and behavioural strategy changes in the rat. We found that on tasks with a forced change in reward criterion, strategy-related representational transitions in mPFC occurred after animals learned that the reward contingency had changed, but before their behaviour changed. On tasks in which animals made their own strategic decisions, representational transitions in mPFC preceded changes in behaviour. These results suggest that mPFC does not merely reflect the action–selection policy of the animal, but rather that mPFC processes information related to a need for a change in strategy.

## Abstract (web summary)

The medial prefrontal cortex (mPFC) is involved in changing behavioural strategies. Recording neural ensembles in rats, Powell and Redish find that the requirement for those changes is represented in mPFC before they manifest behaviourally, both in tasks that externally force a change and in tasks with self-determined change.

## Graphical abstract

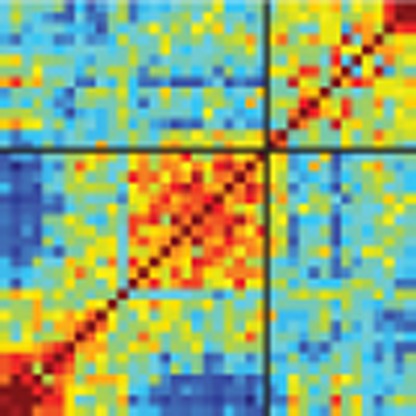

The ability to use different strategies or schemas to deal with changing environments is an essential aspect of intelligent behaviour. Current models suggest that high-level abstractions, removed from physical inputs and outputs, are the key to schema representations^1^,^2^,^3^,^4^. Studies in humans have suggested that aspects of prefrontal cortex (PFC) are critical for strategizing^5^,^6^,^7^,^8^,^9^. In particular, PFC in humans has been identified as critical for changing strategies, such as in the Wisconsin card sort task, in which prefrontal circuits become active during the hypothesis testing of strategies that occurs during strategy shifting^10^. Studies manipulating the rat homologue of PFC^11^ (medial PFC; mPFC) suggest that it has a role in recognizing and changing strategies^12^,^13^.

Indeed, recordings from both non-human primates and rodents have revealed that prefrontal neurons represent the highly abstract information with mixed selectivity needed for these abstract schemas^14^,^15^,^16^,^17^,^18^. In rat, studies have indicated that neurons in prefrontal cortical areas^11^ (mPFC, including prelimbic^16^,^19^,^20^, infralimbic^16^,^21^,^22^ and the anterior cingulate cortex^23^,^24^) reflect ongoing strategies used by the animals to solve behavioural tasks. A structure that is involved in changing strategies should change representations to those predictive of the change in situation-action pairings. However, to date, studies have only compared the representations in mPFC before and after a change in the reward structure governing the task that the animal was performing, and have not looked at the timing of these changes in representation^14^,^15^,^16^,^24^,^25^. A change in the task rules should produce a corresponding change in strategy, and thus a corresponding change in strategy representations. However, if prefrontal representations are critical for the development and implementation of active strategies, then they should also predict changes in strategy even in tasks in which animals make their own (covert) strategy decisions.

To determine the relationship between changes in prefrontal strategy representations and strategy decisions, we derived a general method for detecting transitions between representations in neural ensembles, and applied that method to ensembles recorded from rodent mPFC. We then used this method to uncover latent representation transitions on two behavioural tasks: a spatial reversal task (‘the multiple-T, left/right/alternate' or MT-LRA task) in which rats were forced to change strategy because of a change in the underlying reward-delivery contingencies; and a delay-discounting (DD) task in which strategy changes were covert and internally driven.

## Results

**Figure 1:**
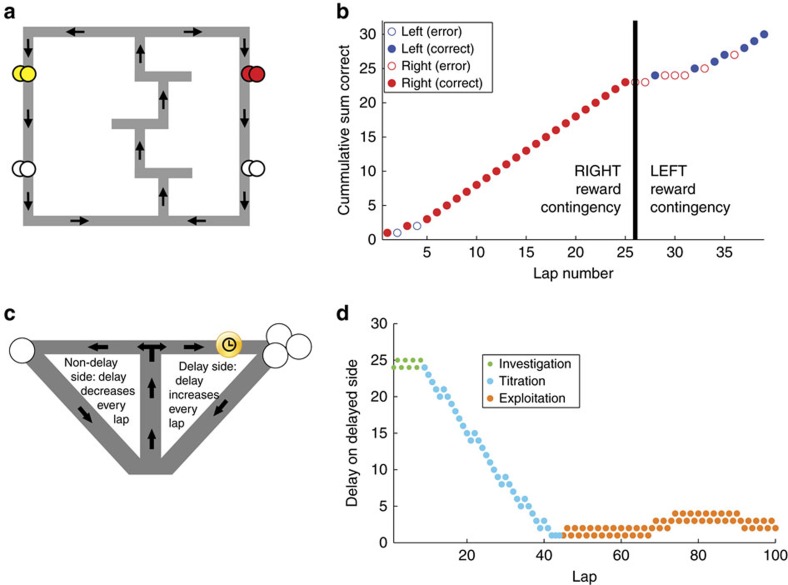
Two tasks with strategy changes. (**a**) The MT-LRA task consists of a central track containing three low-cost T-choices, a high-cost T-choice at the top of the central track and two return-rails on which reward was delivered under either leftward, rightward or alternating contingencies. (**b**) On switch-days, the task contingency changed ∼20 min into the session. On this session, right laps were rewarded in the first part and left laps in the second. Rats were not removed from the track when the reward contingencies switched. See ref. 18. (**c**) The DD task consists of a repeated choice between larger-later and smaller-sooner options. The delay to the larger-later side was adjusted based on the rat's behaviour. (**d**) Rats typically showed three phases on the DD task, alternating to determine which side was delayed and what the starting delay was, titrating the delay by preferentially selecting one side or the other and then exploiting that delay by preferentially alternating between the two options, holding the delay constant. For analysis, neurophysiological relationships to behaviour was measured against six spatial sections on each task (Supplementary Fig. 1).

On the MT-LRA task (Fig. 1a,b), rats ran a loop through four T-choices, the fourth of which led to two return rails on which rats could receive food. Rats received reward under one of three reward-delivery contingencies: left; right; or alternation. Rats were trained to expect one reward contingency (randomly chosen between left, right and alternation) each day. Once the rats were well trained on the one-contingency-per-day version of the task, we began a probe sequence in which the reward contingency changed approximately halfway through each session^18^,^26^,^27^,^28^. Importantly, although animals had never seen this switch in reward contingency before, they recognized this switch and changed their behaviour to a strategy appropriate to the new reward criterion within a few laps^18^,^26^,^27^,^28^. The MT-LRA days studied here thus forced the animal to change strategies in response to an externally driven change in reward criteria.

On the DD task (Fig. 1c,d), rats ran a loop with a single T-choice leading to food reward sites. On this task, animals faced a spatial choice on each lap—one side provided a small immediate reward (smaller-sooner, providing one 45 mg pellet after 1 s) while the other side provided a large reward (larger-later, providing three pellets after a delay *D*). The delay *D* was adjusted based on the rat's choices, allowing the animal to titrate the delay to a preferred choice. Animals on this task typically alternated for a few laps to assess the initial delay (investigation), then adjusted the delay by preferentially selecting one option over the other (titration), and then alternated between sides, which keeps the delay at the same value (exploitation)^29^. Animals thus show strategy changes on the DD task, even though the reward contingency rule does not change.

### Behavioural changes

**Figure 2:**
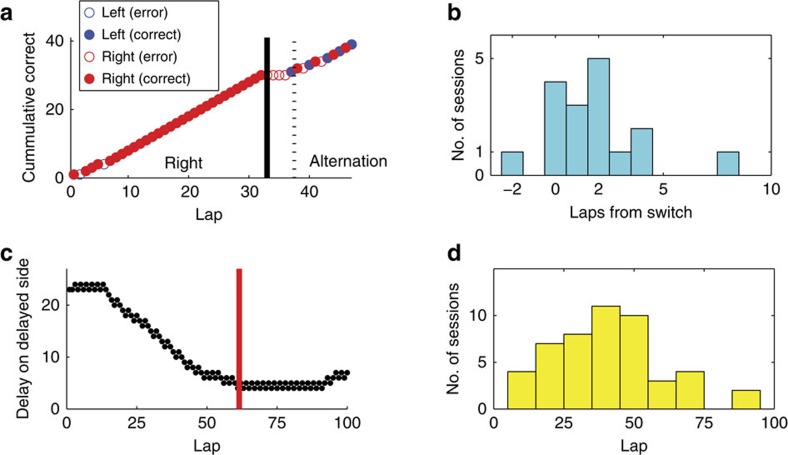
Behavioural change points. (**a**) An example switch session, showing the time at which the reward contingency changed (solid line), and the time the animal's behaviour changed (dashed line). Lap meaning as in Fig. 1b. This session was a left to alternation day. (**b**) Histogram of time between the information switch and the animal's behaviour change. In all but one session, the primary behaviour change occurred after the information switch. (**c**) An example DD session, showing the time of detected behavioural change between the titration and exploitation phases. (**d**) Histogram of lap on which the behavioural change between the titration and exploitation phases occurred.

On the MT-LRA task, animals become informed that the reward-delivery contingency rule has changed when they no longer receive reward after a choice. Over the subsequent laps, they have to adjust their choices to reflect a new strategy to achieve reward. We measured the time of behavioural change using a change-point analysis^30^ (Methods). We found that behaviour changed shortly after the change in reward-delivery contingency (median=2 laps, interquartile range=(1,3)) (Fig. 2a,b). We used a similar change-point analysis to determine the transition between the titration and exploitation phases of the DD task. We found that the lap on which behaviour changed from titration to exploitation varied greatly from session to session, occurring at laps across much of the mid-range of the task (median lap=42; interquartile range=(25,51)).

### Detecting strategy changes

**Figure 3:**
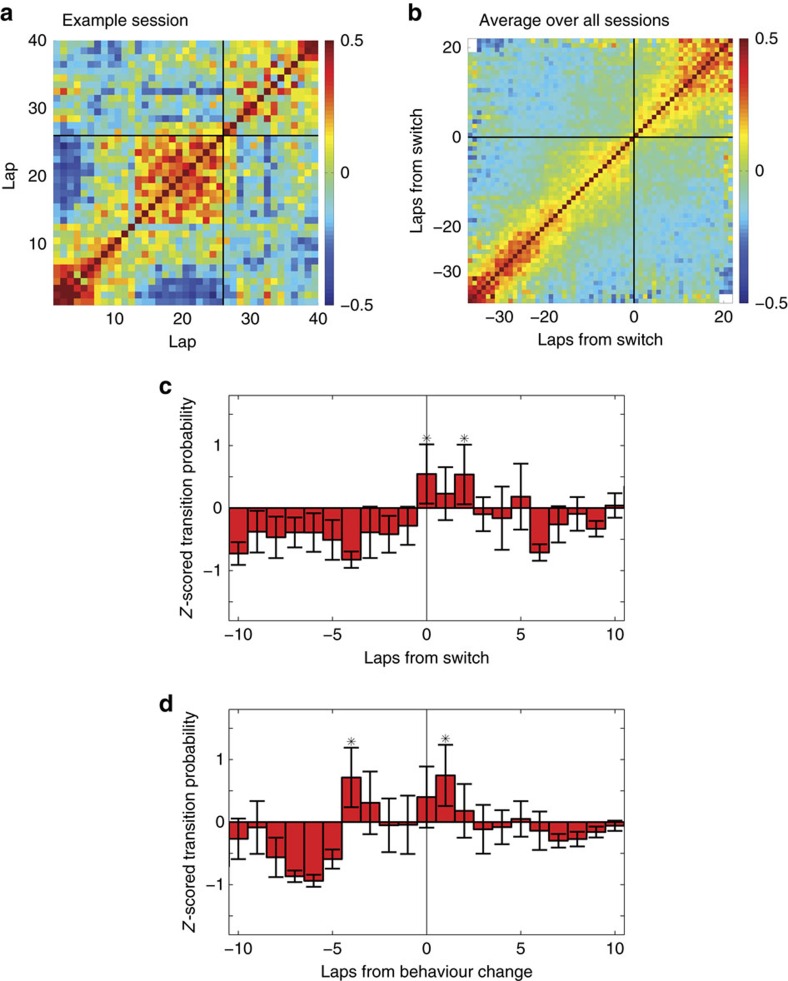
mPFC population firing changes follow task criterion changes but precede behavioural changes on the MT-LRA task. (**a**) Lap–lap correlation of the population firing vectors for one session, with the switch lap marked (black line). (**b**) Average lap–lap correlation of the population firing vectors over all sessions aligned to the switch lap for each session. (**c**) Averaged *Z*-scored transition scores between matched-choice laps over all MT sessions aligned to the switch lap for each session (error bars show s.e.m., *n*=12). Normalization is bootstrap against interspike interval shuffles. (**d**) Averaged *Z*-scored transition scores aligned to the detected behavioural change (error bars show s.e.m., *n*=12). Asterisks indicate values significantly larger than zero (*α*=0.05) as indicated by a *Z*-test. Alternate normalizations are shown in Supplementary Fig. 4.

**Figure 4:**
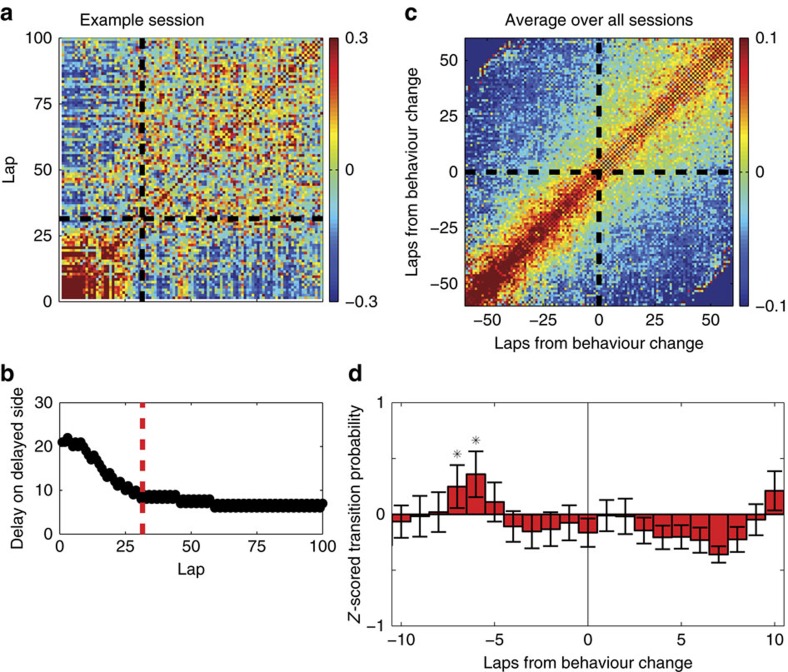
mPFC population firing on the DD task reveals a latent strategy transition. (**a**) Lap–lap correlation of the population firing vectors for one session, with the detected behavioural change lap marked (dotted line). (**b**) The animal's behaviour on that session plotted as delay value by lap, with the behavioural change marked with a red line. (**c**) Lap–lap correlation of the mPFC population firing vectors aligned to the lap of detected maximal behavioural change. Although no clear transition can be seen, our transition-detection algorithm was able to find a clear transition ∼5–8 laps before the detected behavioural change (**d**). Asterisks indicate values significantly larger than zero (*α*=0.05) as indicated by a *Z*-test. Error bars show s.e.m., *n*=45. Alternate normalizations are shown in Supplementary Fig. 5.

Prefrontal cortical cells show a general response to the structure of the task, with cell firing typically reflecting large spatial (task-related) zones^16^,^18^,^31^. Strategy effects occur due to modulations of these overall responses. We therefore followed the methodology of refs 32, 33, in which the main effects driving prefrontal tuning curves was factored out before detecting secondary modulations of those main effects. Thus, to detect representational changes in the population firing representation in mPFC, we factored out the main effects and measured firing rate relative to six task-related spatial zones in the task (Supplementary Fig. 1). We then constructed a population firing pattern vector for each lap and correlated these population firing pattern vectors across the session (Supplementary Fig. 2). Representational correlations showed distinct and abrupt transitions between areas of heightened local correlation that were poorly correlated with each other, indicating transitions between different representations in mPFC (Figs 3a and 4a).

To quantify the tendency of these transitions to occur on any given lap, we created a transition score based on changes in the population firing vector patterns (Methods and Supplementary Figs 2 and 3). Transition scores for each session were *Z*-scored against a bootstrap (*n*_boot_=400) obtained by shuffling the interspike intervals of each cell for each session. This *Z*-scored transition score represents how much more likely a transition was to occur than would be expected from random spiking data with similar spiking characteristics. To correct for the possibility that the representational state in PFC is affected by the physical path the animals are running, we calculated the transition scores for each individual session using only path-matched laps. Alternate normalizations and data without this lap-matching correction are shown in Supplementary Figures.

### Detecting forced strategy changes

To determine when and how the mPFC population representation changed in the face of a forced-strategy change, we trained three rats on the MT-LRA task. If the transitions are reflecting a strategy change, we would expect the transitions to only occur after the information that a strategy change was needed. Across all sessions, the transition scores increased shortly after the switch (Fig. 3).

Even though the average lap-by-lap correlations of the population firing vectors (Fig. 3b) showed a smooth transition over a couple of laps around the switch point, there was still a noticeable transition between distinctly self-correlated states at the aligned switch point. Individual sessions showed a more pronounced transition between these states (Fig. 3a) that often appeared slightly shifted relative to the imposed change in reward contingency, suggesting that the smooth transition in the average plot is due to averaging offset sharp transitions. To directly measure the timing of this sharp change in representation, we measured the likelihood of detecting a transition in prefrontal representations (Supplementary Fig. 3). We compared these transitions with the behaviour-change laps determined earlier. Aligning the average transition scores to this indication of maximal behaviour change indicated a tendency for the transition score to occur several laps before the behaviour change (although there was also a peak on the lap of behavioural change, which may indicate a second change in the representational state as the animal's behaviour starts to match the new strategy).

Representational transitions occurred after the information that a change was needed, but before the actual change in behaviour. This timing suggests that the representational change in mPFC ensembles reflects a process of strategy change. On the MT-LRA task, such a change occurred when animals were forced to change their behavioural strategy to accommodate a change in reward-delivery contingency.

### Volitional strategy changes on the DD task

To examine volitional changes in strategy, we trained three additional rats on the spatial adjusting DD task (Fig. 1c,d), which translates the adjusting DD task^34^ into a spatial T-choice^29^.

It is not necessary for the three behavioural phases (investigation, titration and exploitation) typically seen in each session of the task to occur through different strategies. Economically, for example, a rat could simply express a hyperbolic discounting function^34^,^35^ and always select the most valuable choice given that discounting function. However, current theories of rat decision-making^36^ suggest that these behavioural phases likely access different policies of action selection^29^ and would thus encompass different strategies. Given the known differences between flexible and habitual behaviours in the rat^37^,^38^,^39^,^40^,^41^ and the fact that alternation is a preferred habitual strategy in rats^42^, it is likely that the observed titration and exploitation phases reflect different strategies^29^. Because of the short duration of the investigation phase and the limitations of our detection method (Methods), it would be difficult to identify covert transitions between the investigation and titration phases. However, our strategy representation transition detection method can identify whether or not there are changes in the mPFC representation that reflect the transition between the titration and exploitation phases. Importantly, any strategic transition between the titration and exploitation phases is a covert (volitional) one—no external cues are provided to inform the rat when to transition between strategies.

Figure 4a shows a lap-by-lap population firing vector correlation for an example session, revealing a clear transition between two representational states in mPFC around lap 26. Using a change-point analysis applied to the rat's behaviour^30^, we determined the lap of greatest behavioural change, which for this session occurred around lap 31. To quantify this effect across all rats, we calculated a transition score for all sessions as described above for the MT-LRA task (Supplementary Fig. 3f–j), and found the lap of greatest behavioural change for all sessions for which there were differentiable titration and exploitation phases. The average population firing vector correlation aligned to this lap is shown in Fig. 4c. Heightened probabilities of transition occurred several laps before the behavioural transition (Fig. 4d). This was not a consequence of a large transition probability from a single session or a single animal, as this transition was observed across multiple sessions and multiple animals. Thus, across animals, a change in population representation in mPFC preceded the change in the animal's behaviour, even on volitional tasks in which the change in behaviour was not driven by explicit extrinsic factors.

Because rat PFC has been implicated in motoric behaviours^43^,^44^,^45^, we controlled for the physical path of the animal by only using matched paths in Fig. 4d. Strategies on the DD task include identical paths under different strategy conditions. Note, for example, the repeated lap that drives down the delay in lap 46 (Fig. 4b) and seems to recall the earlier representation (as evidenced by high correlation with early laps), but also note the repeated lap that drives down the delay in lap 59 but does not recall that earlier representation (correlation with early laps remains low). To ensure that we are controlling for physical effects^43^,^44^, Fig. 4d only includes matched paths.

### Behaviour during the transition period

On the MT-LRA task, in the context of a forced-strategy change, mPFC representations changed after the rat learned that the reward contingency changed, but there was a several lap delay before the behaviour decision process changed. Similarly, on the DD task, in the context of a voluntary strategy change, mPFC representations changed several laps before the behaviour changed. An interesting question is what happens between these changes in mPFC representation and the actual change in strategy?

The mPFC is certainly not the only brain structure involved in executing behaviour on these tasks, and it is unlikely to be the most immediate structure that determines behaviour on a lap-to-lap basis. Indeed, animals with mPFC lesions can perform most tasks successfully, and the most common behavioural deficits seen involve switching between behavioural strategies and inhibiting behaviours^46^,^47^. We therefore would assume that a full explanation for the delay will involve knowing how neural representations change in other structures.

To examine whether we could detect behavioural effects during the transition period, we examined vicarious trial and error (VTE) behaviours during this period. When rats come to difficult choice points, they sometimes pause and orient back and forth before making a decision; this process was termed ‘VTE' when it was first observed in the 1930s (refs 48, 49, 50). These behaviours occur after forced strategy changes^18^,^27^,^28^,^51^ and during flexible behaviours^29^,^51^,^52^. Neural recordings during VTE indicate that it reflects an indecisive, deliberative process of exploration of multiple outcomes^53^,^54^,^55^,^56^. The hippocampus represents sequences leading to the potential outcomes^53^,^56^ and the ventral striatum represents the value of those potential outcomes^55^,^57^. Prefrontal disruption leads to a diminishment in VTE behaviour^58^.

**Figure 5:**
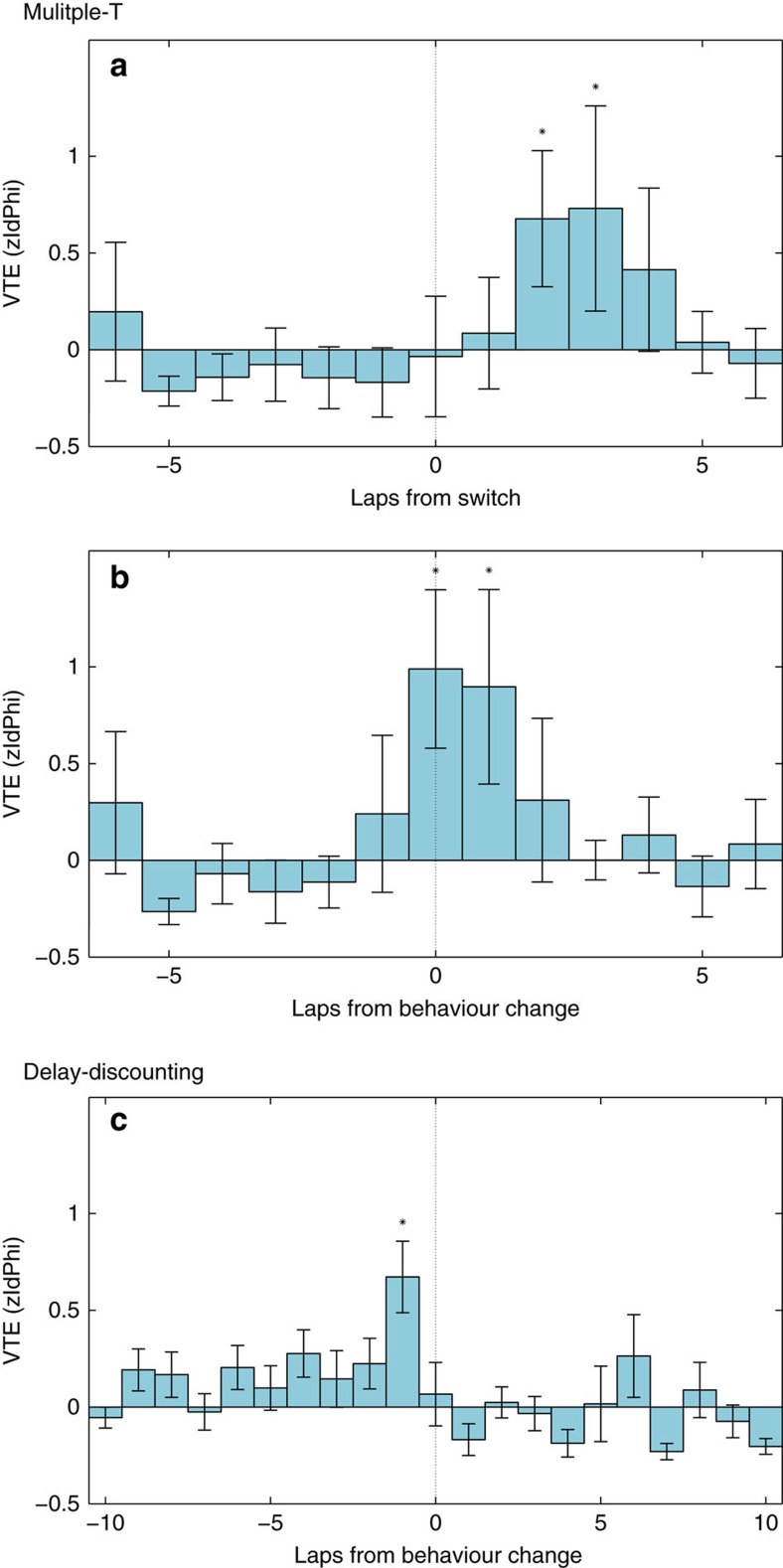
VTE behaviours. When rats come to difficult choices, they sometimes pause and re-orient back and forth before making their decision. This behaviour, termed VTE, is indicative of indecision and the flexible decision-processes necessary to accommodate it^50^. VTE can be quantitatively measured with the *Z*-scored integrated absolute angular velocity through the choice point (zIdPhi, see Methods). (**a**) VTE is significantly increased after the forced strategy-switch on the MT-LRA task. (**b**) VTE is significantly increased around the moment of behavioural change on the MT-LRA task. (**c**) VTE is significantly increased before the moment of behavioural change on the DD task.

VTE behaviour can be quantitatively measured as the integrated absolute angular velocity through the choice point (zIdPhi, see Methods)^28^,^29^. We found that VTE was significantly increased after the switch as the behaviour changed on the MT-LRA task (Fig. 5a,b) and just before the behavioural change on the DD task (Fig. 5c). This suggests that the change in mPFC representation is driving a change in the decision process that is reflected in an increase in VTE behaviours.

### An initial strategy on MT-LRA

**Figure 6:**
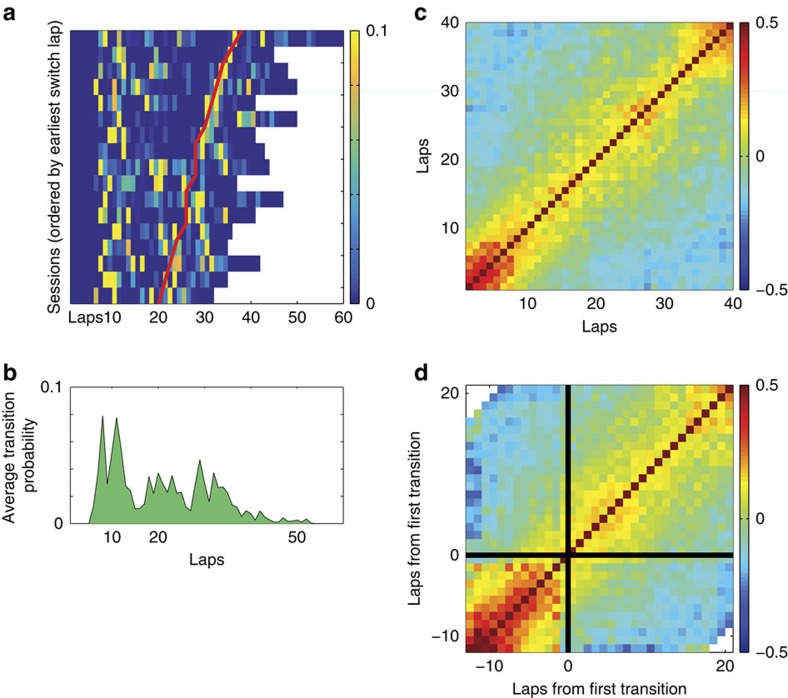
A transition tended to occur after the first few laps on MT-LRA sessions. (**a**) Transition scores of each lap for each session aligned to the start of session, and ordered by the session with the earliest switch lap. We cannot assess transition scores in the first five laps of a sessions, so these first laps are represented as zero. Red line indicates the switch Lap on each session. (**b**) Average transition scores of all sessions shown in **a**. (**c**) Lap-by-lap correlation matrix of population firing averaged over all sessions, aligned to start of session. (**d**) Lap-by-lap correlation matrix of population firing, averaged over all sessions, aligned to lap of highest transition score in the first 15 laps of each session.

**Figure 7:**
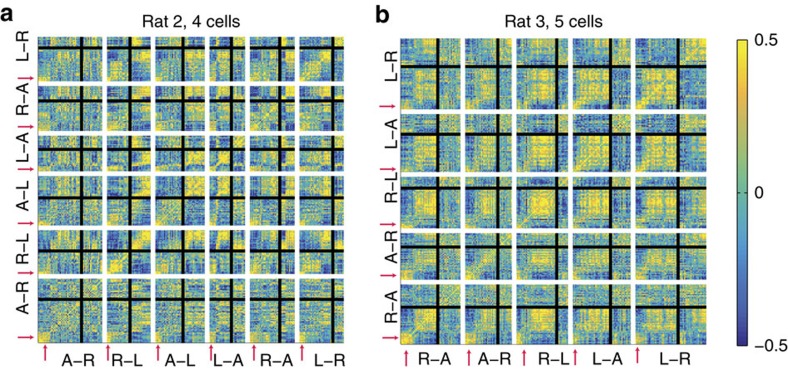
Correlation plots for the spatial population firing vector patterns for cells recorded across several days for two rats on the MT-LRA task. Four cells were recorded across 6 days for rat 2 and five cells were recorded across 5 days for rat 3. White lines separate the individual sessions, and black lines indicate the switch in each session. Note the areas of heightened self-correlation at the beginning of each session that are correlated across sessions.

When the lap–lap population vector correlations were aligned to the start of each MT-LRA session, there was a notable tendency to find a transition before the fifteenth lap of the session. The average of transition scores plotted in Fig. 6 confirmed an elevated transition probability before lap 15. The timing of this transition suggests that perhaps animals entered some preparatory state for the first several laps on the track before switching into a new state consistent with the strategy before the switch. However, it is also possible that animals merely represented one of the three strategies (left, right or alternation) at the beginning of the session before transitioning to another after several laps. To determine if the representational state of the first several laps of each session was consistent across multiple sessions, we took advantage of previous research in which we found that cells recorded across multiple days on this task tend to perform consistently across those daily sessions^18^. Sufficient cells recorded across days were available from two animals (four cells recorded over 6 days from rat 2, five cells recorded over 5 days from rat 3) to calculate correlations across days. Figure 7 shows the lap-by-lap ensemble vector correlations, using only cells recorded for multiple days.

On both of these plots, there was a clear region of heightened correlation for the first several laps of every session. These ensembles had similar relationships between firing rate and behaviour (and thus high ensemble-vector correlations) in the early laps across days; however, these laps were not well correlated with any other laps, indicating that the first several laps of each session formed a representational state consistent with the first several laps of every other session.

The transition from this consistent initial state at the beginning of each session occurred independently of overt changes in the animal's behaviour and independently of any overt change to the task parameters such as the reward criterion. What informational change this covert representational change reflects remains unclear, but it suggests the presence of an initial exploratory phase on the MT-LRA task, perhaps as the animal settles into a specific strategy or as the animal transitions from deliberative to procedural strategies^54^.

## Discussion

Impaired prefrontal function has long been associated with problems in changing behavioural strategy^5^,^6^,^7^,^9^,^10^. Neural recordings from prefrontal areas have been seen to change under strategic changes^8^,^16^,^20^,^23^. If the prefrontal areas are involved in the detection of the necessity of switching strategies and in the switching of the strategy itself, then we would expect to see neural ensemble changes preceding behavioural changes. We developed a novel method of identifying ensemble representation changes and used this new method to identify when prefrontal ensembles changed representation on tasks with strategy changes. We found that the detected changes of ensemble states in rat mPFC followed overt information that a strategy change was necessary, but preceded the onset of behavioural changes reflecting that strategy. Similarly, we found that detected transitions in mPFC ensemble representations preceded behavioural changes for volitionally driven strategy changes as well.

Importantly, the recognition that mPFC encodes a change in strategy does not necessarily imply that it encodes the specific choices made by the animal. In fact, mPFC neural representations have generally been found to have broad task-related activity reflecting sub-parts of the task^16^,^18^,^31^,^59^ or different strategies within tasks^23^,^24^,^60^ often with mixed-selectivity responses reflecting abstract schemas^14^,^15^,^16^,^17^,^18^. Instead, it is possible that mPFC provides additional dimensions to neural representations in other structures to allow those other structures to separate new action-selection policies^9^,^61^,^62^,^63^,^64^. The fact that mPFC representations change before behaviour further implies that mPFC is unlikely to be directly driving the action-selection choices of the animal, because the mPFC representations were different even though the behaviour remained the same. Our data suggest the mPFC provides signals that facilitate changes in behavioural strategy.

Recent functional magnetic resonance imaging data from human subjects suggest a similar process occurs in human prefrontal areas^4^, and support the conclusion that representations in mPFC change significantly before behaviour. Schuck *et al*. linked the representational changes they detected to enhanced representations of certain task relevant stimuli that were essential for one strategy but extraneous for the other. Although it is possible that a change in sensitivity to task-related stimuli may play a role in our ensemble firing pattern changes, and there is ample evidence that single cells in PFC are sensitive to individual task stimuli^18^,^31^,^65^,^66^, our tasks were designed to eliminate any such stimuli, unlike the task used by Schuck *et al*.^4^ in which the change in strategy was prompted by using previously ignored stimuli. The representational changes we report in rat mPFC were not prompted by animals visiting different areas of the track not commonly visited in the other strategy, nor were there any explicit cues that would inform the rat of the appropriate strategy. Instead, the strategy changes reflected proportional changes in action-selection processes on these tasks.

The rat mPFC has motoric outputs and motoric relationships^43^,^44^,^45^,^67^,^68^. This has led to a concern that has come to be known as the Euston–Cowen–McNaughton Hassle^43^,^44^. The Euston–Cowen–McNaughton Hassle entails the observation that subtle behavioural differences have been shown to be able to account for some previous attempts at identifying strategy representations in rodent mPFC. To ensure that we have compared motorically similar laps with each other, we compared path-matched laps (Methods, Supplementary Figs 6 and 7. An important advantage of the DD task is that the different strategic components are not executed with different behavioural patterns. During the titration strategy, the animals predominantly chose one side of the track or the other, but they also made laps to the other side. (See Fig. 1d, in which the downward titration was punctuated by several alternation laps temporarily holding the delay constant.) Similarly, during the exploitation strategy, the animals predominantly alternated (holding the delay constant), but they also occasionally made laps to the same side. (See Fig. 1d, in which there were occasional steps upward or downward during the exploitation strategy.) On the DD task, there were four different paths available to the animals, determined by their start and end locations on a given lap: right to left laps (RL); left to right laps (LR); right to right laps (RR); and left to left laps (LL). Because any of these paths can occur within any strategy (titration is predominantly, but not exclusively LL or RR; exploitation is predominantly but not exclusively alternating LR/RL pairs), rats showed examples of each path in each strategy phase. Our analyses were based on comparisons of transitions between representations for matched-path laps. The population firing pattern vector of laps within a specific strategy was more strongly correlated with other laps within that strategy than similar-path laps during other strategies, indicating that the population transitions were more likely due to overall strategic states rather than individual lap types (Supplementary Figs 6 and 7)

As additional evidence that changes in cues were not responsible for a state change in PFC, consider the initial state discovered on the MT-LRA task. There are no overt cue differences in the first several laps of the task that could account for this state, and although it is possible that animals were paying attention to different aspects of the task after the first several laps, this explanation seems unlikely since these animals had been running the same task for many weeks by the time these data were recorded in a room with controlled cues. We can only speculate about the cognitive nature of this initial state, but the fact that it was consistently represented across several days of recording and dissipated after the first few laps suggests that it most likely represents a ready state for performing the task in general before the animal transitions into a specific representation of the behavioural pre-switch strategy. There was some evidence for a similar state in some DD sessions (data not shown) but its presence was not as clear or consistent as on the MT-LRA sessions.

Strategy can be defined as the action-selection policy^69^. A strategy representation is thus one which identifies the action choices for a given situation. By examining the relationship between representational state transitions in PFC and changes in the animal's behaviour, we identified changes in representations that reliably preceded strategy changes. Following strategy changes forced on the animal from outside signals (by changing the reward-delivery contingencies), prefrontal changes followed the animal's receipt of information that reward-delivery contingencies had changed but preceded the consequential behavioural changes. On volitional strategy changes, representational changes preceded the observable behavioural changes. These representational transitions preceding behavioural changes suggest that the rodent mPFC does not merely reflect the action-selection policy of the animal, but instead encodes the necessity of making strategic changes and the new strategic changes needed.

## Methods

### Animal training

All experiments described in this paper were carried out on male Fisher-Brown Norway rats (three for the multiple-T task, three for the DD task, six in total) aged 8–12 months at the start of behaviour. These animals were housed on a 12 h light–dark cycle and all experiments for a given rat were run at the same time every day during the light phase. Animals were handled and trained to eat flavoured food pellets (45 mg each, Research Diets, New Brunswick, NJ, USA) for 1 to 2 weeks before the start of task training. These same food pellets were used as a reward on all tasks. To motivate task running, animals were deprived of food in their home cages during their period of task running, but were always given free access to water in their home cages. Animals were weighed daily and were returned temporarily to free food access if their weights dropped below 80% of their initial free food weight or if they displayed any signs of illness. All training procedures were approved by the Institutional Animal Care and Use Committee at the University of Minnesota, and were in accordance with the National Institutes of Health guidelines.

### Surgery and recordings

Neural data from all rats were recorded on a 64 channel analogue Cheetah recording system (Neuralynx, Bozeman, MT, USA). Spikes were sorted offline by first separating them into putative clusters using KustaKwik (KD Harris) and then individually verifying and adjusting those clusters with the MClust 3.5 Software package (A.D.R.). All spiking activity was co-registered to the animal's behaviour and task events recorded with custom written programmes in Matlab (The Mathworks, Natick, MA).

A hyperdrive containing 12 tetrodes was surgically implanted into each animal using well-established surgical procedures. Animals were anaesthetized with sodium pentobarbital (Nembutal, 50 mg kg^−1^, delivered intraperitoneally) then placed in a sterotactic frame. During surgery anaesthesia was maintained with isoflurane mixed at 0.5–2% with oxygen and delivered via a nosecone.

The first rat on the MT-LRA task was implanted with all the tetrodes and references grouped in a single circular bundle ∼1.5 mm in diameter. The craniotomy for this surgery was located 3.0 mm anterior of bregma, 1.3 mm lateral of the midline (on the right side) and angled at 9.5° from the vertical towards the midline^18^. All the subsequent rats used an alternate implantation technique with a split bundle design, in which the tetrodes were divided evenly (six tetrodes and one reference each) into two bundles with a 1.0 mm spacing between them. These bundles were then implanted such that one bundle was directly over the PL on either side of the sagittal sinus, 3.0 mm anterior to bregma^18^.

After surgery, animals were given a 3-day course of antibiotics (Baytril) and returned to free food to recover for several days before being returned to task running. No animals were returned to running before their weights had stabilized and they appeared fully recovered from surgery. Tetrodes were turned in small increments every day following surgery until they reached the desired target coordinates. After all data had been collected from each animal, recording locations were verified histologically to be in the inferior prelimbic cortex.

### The multiple-T left, right, alternation task

The MT-LRA task and associated behaviour has been described in detail elsewhere^18^,^26^,^27^,^28^.

The MT-LRA task was run on an elevated sideways-8 shaped maze (Fig. 1a). On a single lap, rats started from the start of maze (SoM) region at the bottom of the track, and ran through a navigation sequence that consisted of three low-cost choice points to a high-cost choice point region (CP) at the top of the track. The low-cost choice points consisted of T-shaped regions that were pseudo-randomly arranged each day so that the animal would have to re-learn the sequence of turns. At the high-cost choice point the animal had to choose whether to proceed to the left or to the right. Either side would take him past two feeder locations, where pellets would be dispensed automatically if he made the correct choice, and then back around to the SoM region again to begin another lap. We referred to the region of the track between the CP and the feeder zones as the top rail (Top), the area around and between the feeders as the feeder zone and the area between the feeders and the SoM as the bottom rail (Bot).

At the beginning of a session, animals were placed on the track at the SoM location, then allowed to continuously run as many laps as possible for 40 min. Each successful lap resulted in the automated delivery of two reward pellets at each of the two feeder locations encountered, for a total of four pellets for each successful lap. The first feeder location on the left side of the track dispensed banana-flavoured food pellets, and the first feeder location on the right side of the track dispensed berry-flavoured food pellets. The second feeder location on either side dispensed unflavoured, white-coloured food pellets.

There were three different reward criteria that specified the correct decisions on each lap, left reward (L), right reward (R) and alternating reward (A). For the left and right reward criteria the animal was rewarded for running only to the left or right sides of the track, respectively. In the alternating reward criterion, the animal received reward if it went to the side opposite to the one most recently rewarded (under the alternation condition, the first lap of a session was always rewarded). During all training days, animals experienced only one pseudo-randomly chosen reward criterion per day. However, once animals were fully trained and behaving well, we began the 6-day switch sequence. During this period, animals began each session with a single reward criterion as with all previous sessions, but approximately halfway through the 40 min session (randomly determined between 18 and 22 min) the reward criterion switched to one of the others. No external cue indicated this change in reward criterion, the animal had to learn it from failed reward incidents. The switch sequence consisted of six days so that all pairs of initial and final criteria were presented. The MT-LRA data reported in this paper are from the switch sequence. Importantly, animals had never experienced a within-session change in reward-contingency before the first of this 6-day sequence.

The expected behaviour on MT-LRA was for animals to rapidly identify the daily strategy over the course of the first several laps, then exploit that strategy reliably (that is, perform laps at a high rate of success) up until the switch. At the switch lap, their behaviour should drop down to perseveration levels (∼1/6 laps correct, the expected rate of continuing to use the now-incorrect strategy) until they recognized their error, identified the new strategy, and returned to performing at a high level over the course of the next few laps. The behaviour of rats in this study reported elsewhere^18^, closely matched this pattern.

### The DD task

The DD task and its associated behaviour has been discussed in detail elsewhere^29^. Here we review the task and the basic features of typical behaviour.

The DD task took place on an elevated T-maze with two return rails that allowed animals to run continuously (Fig. 1c). At the main T junction, animals ran to the left or right sides of the track, each of which had a feeder that dispensed unflavoured (white) food pellets. On one side of the track, the animals received one food pellet after a 1 s delay. On the other side of the track, animals received three food pellets after a variable delay. The location of large and small rewards on the left or right side of the track was consistent within each session, but varied between sessions. The variable delay was initialized at the start of each session to a value pseudo-randomly chosen between 1 and 30 s (all values were sampled only once over the course of a 30-day training session, and were controlled so as to not have too many high or low delays over several consecutive sessions). The variable delay also adjusted based on the animal's decisions, such that it increased by 1 s with every visit to the large reward side of the track, and decreased by 1 s with every visit to the small reward side of the track. This adjustment procedure allowed the animals to vary the delay by making multiple consecutive visits to either side of the track, or to maintain the current delay by alternating visits to each side of the track. All delays were indicated by a tone that counted down with decreasing frequency each second until reward was delivered by the automated feeder system (Med-Associates, St Albans, VT, USA). These tones were standardized so that the same tone always indicated the same number of seconds of delay to wait. Longer delays were indicated by higher frequency tones, with each 1 s increase marked by a 175 Hz increase in pitch. Tones started counting down as soon as the animal reached a delay zone region near the feeder on each side of the track, and continued as long as he remained in this region until food was dispensed at the end of the delay. Animals were free to skip any delays by running past the feeder and on to the next lap (at which point the tones would stop and no food would be delivered). In practice, animals tended to wait out almost all delay periods.

Animals performing the DD task displayed a typical pattern of behaviour (Fig. 1d) over the course of a session, which was typified by three distinct behavioural stages. First, animals would express a brief investigation stage that lasted several laps during which they would determine which side had the large and small reward on a given session, and how long the initial delay was for that session. Then animals typically entered a titration phase, during which they made laps predominantly to one side of the track or the other to drive the delay value to a more preferable length. Finally, once the delay was adjusted so that the overall reward on each side of the track was equal, animals would exploit the equal value of the two sides, by alternating laps to either side of the track, which held the delay constant. They typically continued this exploitation stage until they reached the maximum number of laps (100) or time limit of the session (1 h).

Animals ran daily sessions of either 100 laps or 1 h, whichever elapsed first. Animals ran 100 laps in less than an hour on nearly all recording sessions. The training phase of the task consisted of 30 sessions before surgical implantation so that every initial delay between 1 and 30 s could be sampled. Following recovery from surgery, animals ran another 30 sessions so that again every initial delay from 1 to 30 s could be sampled, while spiking data were recorded from PFC.

### Data analysis: transition score

The transition score represents the probability that the representational state of PFC changed on a given lap of a session. To calculate the transition score we began by calculating the spatial firing vectors for each lap. Our tasks were divided into seven bins, including the six zones mirrored on each side of the track (bins are depicted by colour codes in Supplementary Fig. 1) along with the overall firing rate on that lap. We found the *Z*-scored firing rate for each cell in each spatial bin on every lap, and used these firing rates to create a population code that consisted of an *M*-dimensional vector for each lap that represented the *Z*-scored firing of each cell in each spatial bin (*M*=*N* cells × 7 (spatial bins)). Each of these *M*-dimensional vectors represents the population state of the ensemble of cells recorded from the mPFC for that lap, which is a proxy for the representational state of the mPFC as a whole on that lap. For visualization purposes, we created plots showing the correlation coefficient of each lap's *M*-dimensional vector correlated with every other lap's *M*-dimensional vector. These lap-by-lap ensemble vector correlation plots make it easy to see when a significant change in the state of the PFC population code has occurred.

To automatically determine the likelihood that an abrupt transition between two locally consistent states has occurred on a given lap, we used a *K*-means analysis on the *M*-dimensional population firing vectors. The *K*-means analysis assigns these firing vectors into clusters of laps with similar population firing characteristics. Examples are shown in Supplementary Fig. 3c,h. From the *K*-means fits we can determine the lap on which a transition between different clusters occurred by using a change point analysis on a cumulative sum of the cluster labels over laps^30^ (note, these cluster labels are arbitrarily assigned, but that does not affect the change point analysis. Examples of this analysis is depicted in Supplementary Fig. 3d,i. The laps identified by the change point analysis indicate when a transition occurred. However, because the *K*-means fit depends on the initial allocation of laps to clusters and the selection of a *K* value, we repeated the above analyses 100 times at *K* values of 2, 3, 4 and 5 for each session. We collected a histogram of the number of times a change point was detected for each lap over the 100 iterations (Supplementary Fig. 3e,j). This number is defined as the transition score for each lap of a session.

To make the end results more interpretable, we created two different sampling distributions to compare our actual data with. First, we created a distribution by running the same algorithm on 400 control sessions generated by shuffling the inter-spike intervals of the cells in the ensemble. This distribution provides a null hypothesis of transition scores that would be calculated from random firing data. Second, we used the actual transition scores from each session but randomly assigned the switch lap to any lap in the session. We used 500 random assignments to create the second distribution. We *Z*-scored the actual transition scores to both of these distributions to produce the *Z*-scored transition score data reported in this paper.

### Data analysis: detecting the behavioural change

We used a change point analysis^30^ to determine the lap on which the animal's behaviour changed the most for both the MT-LRA and the DD tasks. Laps were coded 1 for visits to one side (left for MT-LRA and delayed side for DD) and 0 for visits to the other side, then a change point analysis^30^ was run on the cumulative sum of the laps over the course of the session (Fig. 2)

For MT-LRA sessions, the behavioural change point was used to determine when the animal's behaviour changed independent of when the criterion switch actually occurred. On average, the greatest behavioural change on a session occurred 2.3 laps after the switch.

For DD sessions there was no switch lap for comparison so we could only compare cellular firing transitions with empirically determined behavioural change laps. Sessions (*n*=16) on which a behavioural change lap could not be determined were excluded from DD analysis, leaving us with 45 DD sessions. One MT-LRA session did not have a clear behavioural change point, so only 17 MT-LRA sessions were used in behavioural change analyses, although all 18 sessions were used in the switch lap analyses.

Two critical points about the change point algorithm we used need to be addressed. First, change points identified by the algorithm occur between two laps. By convention, the change point is assigned to the following lap, and this convention was used for both behavioural change points described here and for the transition scores assigned above. Similarly, the switch lap for MT-LRA was defined as the first lap on which the animal did not receive food because of an incorrect choice following the switch and marks the lap after a switch has occurred. Second, the change point code requires a baseline behaviour to compare for assessing changes, so it is incapable of determining a change within five laps of the beginning or the end of a session.

### Data analysis: controlling for path difference effects

To ensure that differences in the paths animals run on different laps of the MT-LRA task were not causing the differences we detected in the ensemble firing patterns, we isolated laps run only to a specific side of the track and re-calculated the transition scores using only these laps. For the MT-LRA task we separated out laps run to the left side of the track (from SoM back to SoM) from laps run to the right side of the track, and analysed them separately. For these control analyses, we considered only days in which one of the reward criteria was alternation because on days where the rat switched from the left to right contingency (or vice versa) tended to have too few matchable laps before and after the switch. An example of this exclusion process is shown in Supplementary Fig. 6. Only 12 MT-LRA sessions met these restricted criteria. We calculated the *Z*-scored transition scores as described above, and aligned the results to both the switch lap and the lap of greatest behaviour change.

To control for potential effects of path differences on the DD task, we divided DD laps into four categories: laps that started at the left feeder and ended at the right feeder (LR laps); laps that started at the right feeder and ended at the left feeder (RL laps); laps that both started and ended at the right feeder (RR laps); and laps that both started and ended at the left feeder (LL laps). Because LL and RR laps are rare, but RL and LR laps are common and occur in all three task stages, we were able to calculate the *Z*-scored transition score (described above) for all sessions using only the LR or RL laps from each session. An example of this exclusion process is shown in Supplementary Fig. 7.

### Data analysis: VTE (zIdPhi)

To quantify VTE behaviour through the choice point, we calculated the zIdPhi measure, which we have previously found to provide clear distinctions between straight passes through the choice point and the pause-and-reorient behaviours of VTE. We first calculated the spatial velocity as the first derivative of the 〈*x*, *y*〉 position sequence using the Janabi-Sharifi *et al*.^70^ algorithm, which reduces noise on straight sequences (when the velocity does not change) while still capturing moments of acceleration accurately. We measure the orientation of motion d*ϕ* as the arctangent of the 〈dx, dy〉 sequence, and then calculate the derivative of the orientation of motion 〈d*ϕ*〉 using the Janabi-Sharifi *et al*.^70^ algorithm again. We then integrate the absolute value of 〈d*ϕ*〉 over the choice point pass. Finally, to account for differences between rats and sessions, we *Z*-score the measure. zIdPhi can be used as a continuous measure of the likelihood that we are observing a VTE event on a given pass through a choice point.

### Data analysis: initial state comparison on the MT-LRA task

We examined the heightened correlation on the early laps of MT-LRA sessions by aligning both transition scores and average lap-by-lap ensemble vector correlation plots to the first lap of the session. In addition, we aligned all MT-LRA sessions to the first transition detected in the cellular data. This was defined as the lap with the highest transition score among the first 15 laps of each session.

To determine whether the heightened correlation state at the beginning of each session corresponded to a consistent state across days within the same animal, we created a lap-by-lap ensemble vector correlation across several consecutive days for two rats. In each case, we identified all cells recorded consecutively across several days recording^18^. We found four cells recorded across all 6 days for rat 1, and five cells recorded across 5 days for rat 2. We calculated population vectors as described above using only these subsets of cells for all laps across all days, and correlated these ensemble vectors across all laps.

### Data availability

All data are available from the authors, and will be provided with appropriate exegesis if requested.

## Additional information

**How to cite this article:** Powell, N. J. and Redish, A.D. Representational changes of latent strategies in rat medial prefrontal cortex precede changes in behaviour. *Nat. Commun.* 7:12830 doi: 10.1038/ncomms12830 (2016).

## Supplementary Material

Supplementary Information Supplementary Figures 1-7
