# Supplementary material for: Representational changes of latent strategies in rat medial prefrontal cortex precede changes in behaviour
Source: Nat Commun. 2016 Sep 22;7:12830. doi: 10.1038/ncomms12830 (PMC5036147; doi:10.1038/ncomms12830)
Supplement: Supplementary Information — Supplementary Figures 1-7 [file ncomms12830-s1.pdf]

a

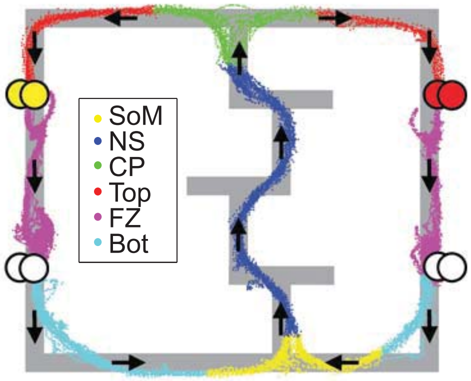

b

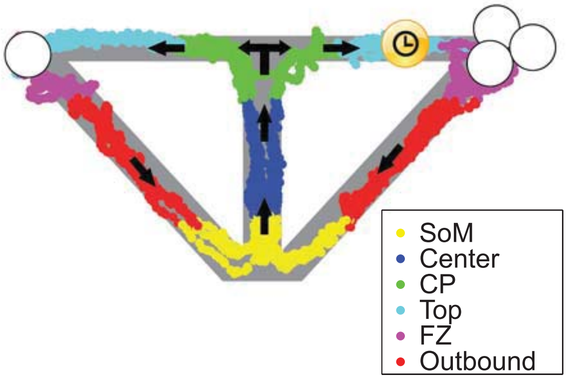

SUPPLEMENTARY FIGURE 1

For analysis, neurophysiological relationships to behavior was measured against six spatial sections on each task. **a**: MT-LRA. SoM=Start of maze. NS=Navigation sequence. CP=Choice point. Top = Top rail. FZ = Feeder zone. Bot = Bottom (return) rail. **b**: DD. SoM=Start of maze. Center = Center rail. CP=Choice point. Top = Top rail. FZ = Feeder zone. Outbound = Starting path from feeder to SoM.

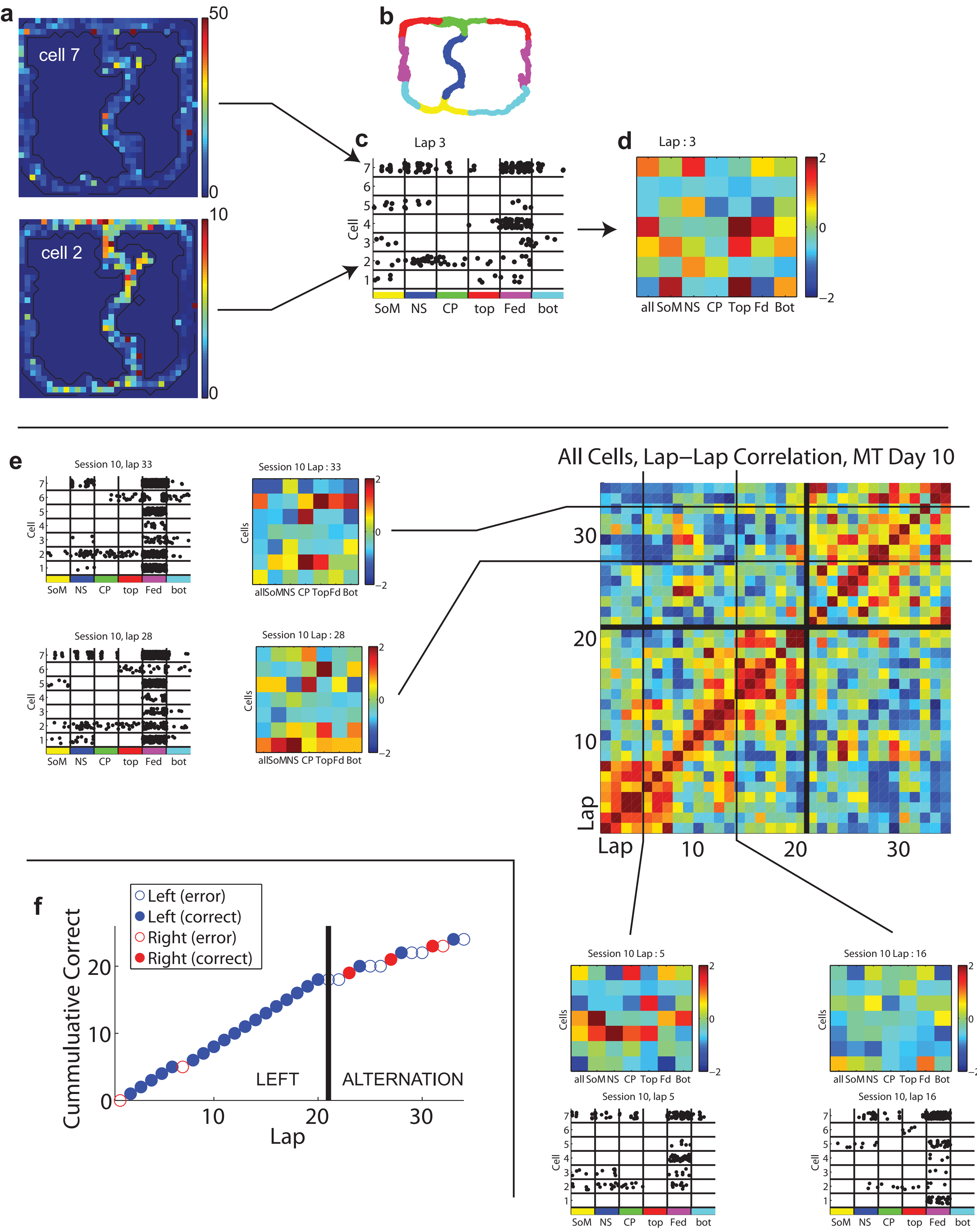
a
cell 7
50
0
cell 2
10
0
b
c
Lap 3
Cell
7
6
5
4
3
2
1
SoM
NS
CP
top
Fed
bot
d
Lap : 3
2
0
−2
all SoM NS CP Top Fd Bot
e
Session 10, lap 33
Session 10 Lap : 33
Cells
allSoMNS CP TopFd Bot
Session 10, lap 28
Session 10 Lap : 28
All Cells, Lap−Lap Correlation, MT Day 10
Lap
10
20
30
Session 10 Lap : 5
Session 10, lap 5
Session 10 Lap : 16
Session 10, lap 16
f
Left (error)
Left (correct)
Right (error)
Right (correct)
Cummulative Correct
LEFT
ALTERNATION
Lap

SUPPLEMENTARY FIGURE 2

Construction of the cross-correlation matrices. a: Cell tuning curves were constructed. Cells have typical firing patterns across the maze, often reflecting specific chunks of the maze. **b**: We categorized this firing into six zones (see also **Fig. S1**). **c**: We formed a matrix of firing of each cell in each zone for a given lap based on the firing rate in each zone, z-scored relative to all laps. See **Methods**. **d**: Factoring the overall firing rate of each cell as an additional column, this gave us an $m \times n$ matrix termed the population firing vector pattern where m was the number of zones (7) and n was the number of cells. **e**: Each $m \times n$ PFVP matrix for each lap was correlated with all the other matrices for each of the other laps, providing a correlation matrix of firing-pattern-similarity between laps. **f**: The example correlation matrix shown in **e** was a left to alternation day.

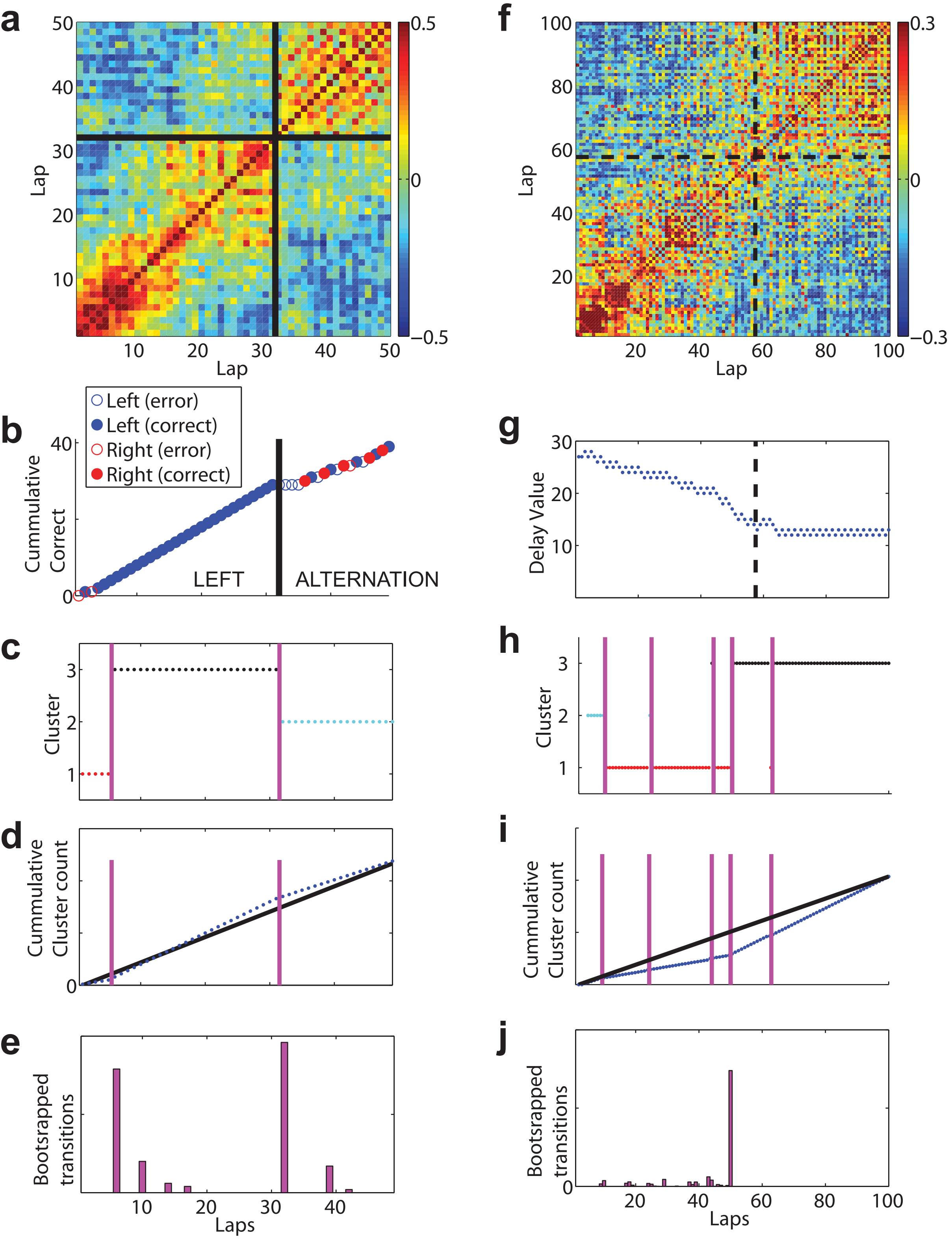
a
f
Lap
Lap
0.5
0
−0.5
0.3
0
−0.3
b
Left (error)
Left (correct)
Right (error)
Right (correct)
Cummulative Correct
LEFT
ALTERNATION
g
Delay Value
c
Cluster
h
Cluster
d
Cummulative Cluster count
i
Cummulative Cluster count
e
Bootsrapped transitions
Laps
j
Bootsrapped transitions
Laps

SUPPLEMENTARY FIGURE 3

Identifying transitions in representations. We first constructed lap-by-lap correlations between population vectors constructed from behavioral correlates of prefrontal firing patterns. (See **Fig. S2**.) We then used K-Means clustering to detect clusters of similarly-represented laps, and measured the changes between those clusters. See Methods for further details. **a-e**: Finding clusters on the MT-LRA task. **a**: Lap-by-lap mPFC representational correlations on a sample session. **b**: Behavioral plot showing left/right choices and correct/error consequences. The reward contingency switch is shown with a black line. **c**: K-Means clustering. **d**: We find the transitions by identifying changes in the clusters. **e**: Because clustering is noisy and depends on the number of clusters chosen, we ran the clustering 100 times with each of 2-5 clusters. Panel **e** shows the number of transitions over these multiple runs. Note the clear increases on laps 6 and 32. (The reward-delivery switch occurred on lap 32.) **f-j**: Finding clusters on the DD task. **f**: Lap-by-lap mPFC representational correlations on a sample session. **g**: The behavioral plot showing delay as a function of lap. Detected behavioral change is shown with a dotted black line. **h**: Sample K-Means clustering. **i**: Identified cluster transitions. **j**: The only consistent cluster transition occurred on lap 50, approximately 7 laps before the detected behavioral change.

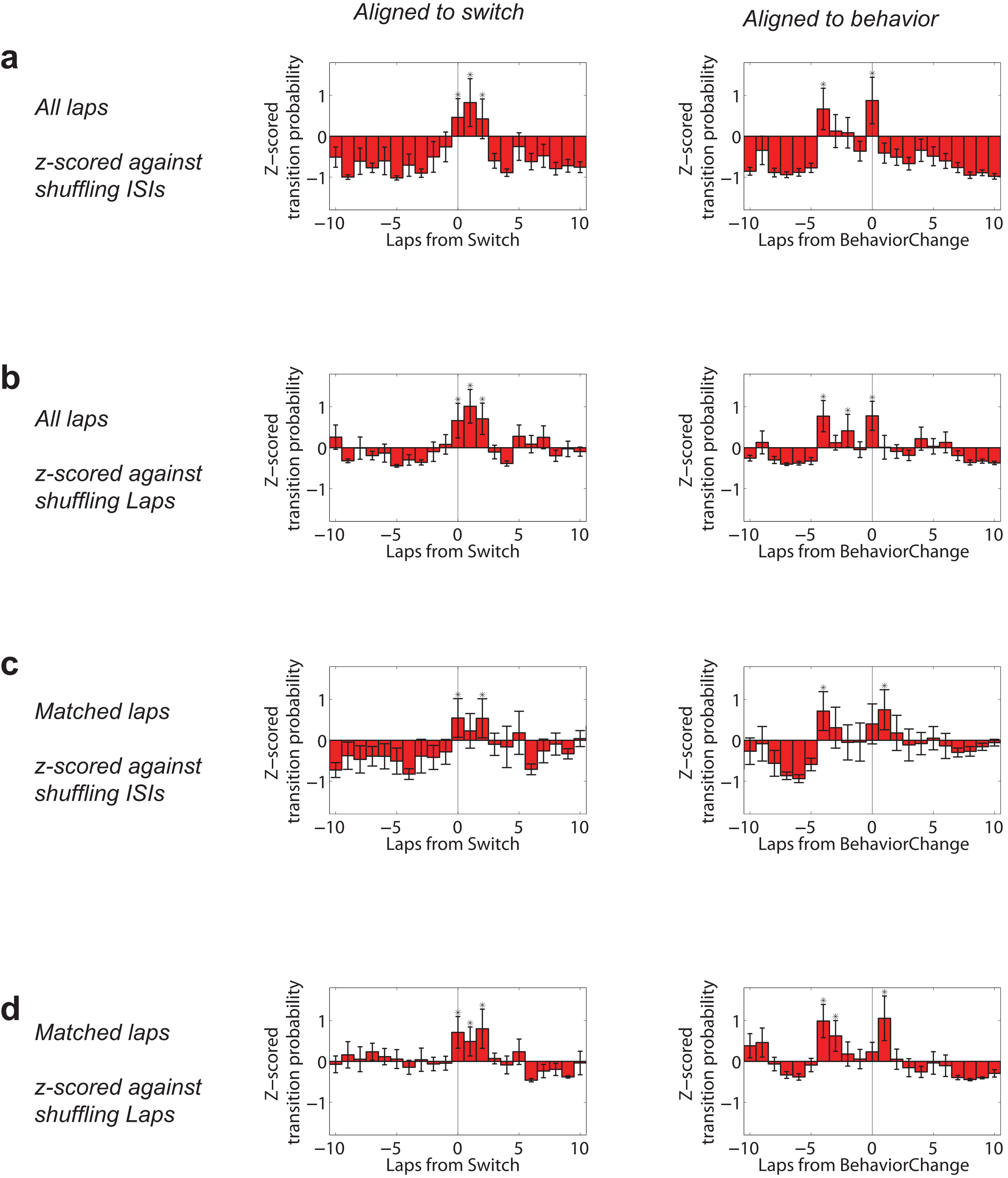
Aligned to switch
Aligned to behavior
a
All laps
z-scored against shuffling ISIs
b
All laps
z-scored against shuffling Laps
c
Matched laps
z-scored against shuffling ISIs
d
Matched laps
z-scored against shuffling Laps
Z−scored transition probability
Laps from Switch
Laps from BehaviorChange

SUPPLEMENTARY FIGURE 4

Alternate normalizations for population transition detection. Each panel shows averaged z-scored transition scores between matched-choice laps over all MT sessions aligned to the switch lap (left) and behavioral lap (right) for each session. Asterisks indicate values significantly larger than zero ($\alpha = 0.05$) as indicated by a ztest. Error bars show SEM, n=17. **a**: Normalization using bootstrap against ISI shuffles. Correlations were done over all laps, ignoring direction. **b**: Normalization using bootstrap against lap shuffles. Correlations were done over all laps, ignoring direction. **c**: Normalization using bootstrap against ISI shuffles. Correlations were done only between matched-direction laps (i.e. left-choice with left-choice and right-choice with right-choice). Laps to different sides did not contribute to the correlations. **d**: Normalization using bootstrap against lap shuffles. Correlations were done only between matched-direction laps (i.e. left-choice with left-choice and right-choice with right-choice). Laps to different sides did not contribute to the correlations.

Aligned to behavior

a

All laps

z-scored against shuffling ISIs

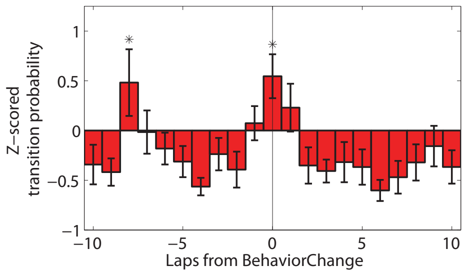

b

All laps

z-scored against shuffling Laps

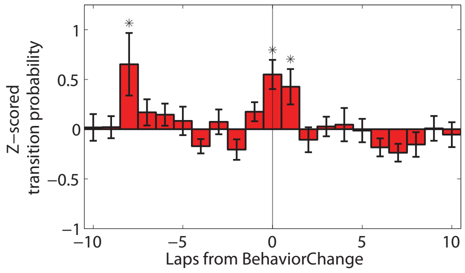

c

Matched laps

z-scored against shuffling ISIs

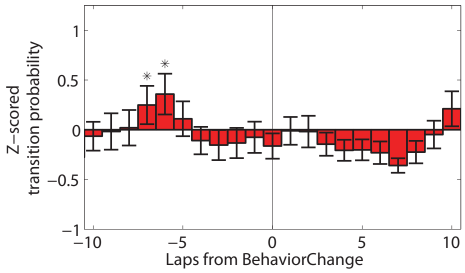

d

Matched laps

z-scored against shuffling Laps

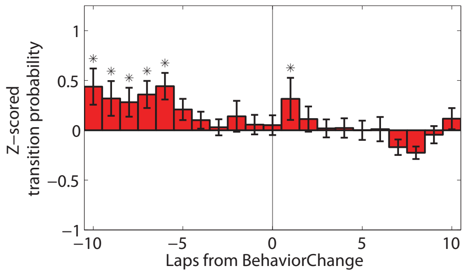

SUPPLEMENTARY FIGURE 5

Alternate normalizations for population transition detection. Each panel shows averaged z-scored transition scores between matched-choice laps over all DD sessions aligned to the lap of identified behavioral change. Asterisks indicate values significantly larger than zero ($\alpha = 0.05$) as indicated by a ztest. Error bars show SEM, n=45. **a**: Normalization using bootstrap against ISI shuffles. Correlations were done over all laps, ignoring direction. **b**: Normalization using bootstrap against lap shuffles. Correlations were done over all laps, ignoring direction. **c**: Normalization using bootstrap against ISI shuffles. Correlations were done only between matched-direction laps (i.e. LR laps matched with LR laps and RL laps matched with RL laps, LL and RR laps were not included because there were too few on any given session to provide reliable estimates). Unmatched laps did not contribute to the correlations. **d**: Normalization using bootstrap against lap shuffles. Correlations were done only between matched-direction laps (i.e. LR laps matched with LR laps and RL laps matched with RL laps, LL and RR laps were not included because there were too few on any given session to provide reliable estimates). Unmatched laps did not contribute to the correlations.

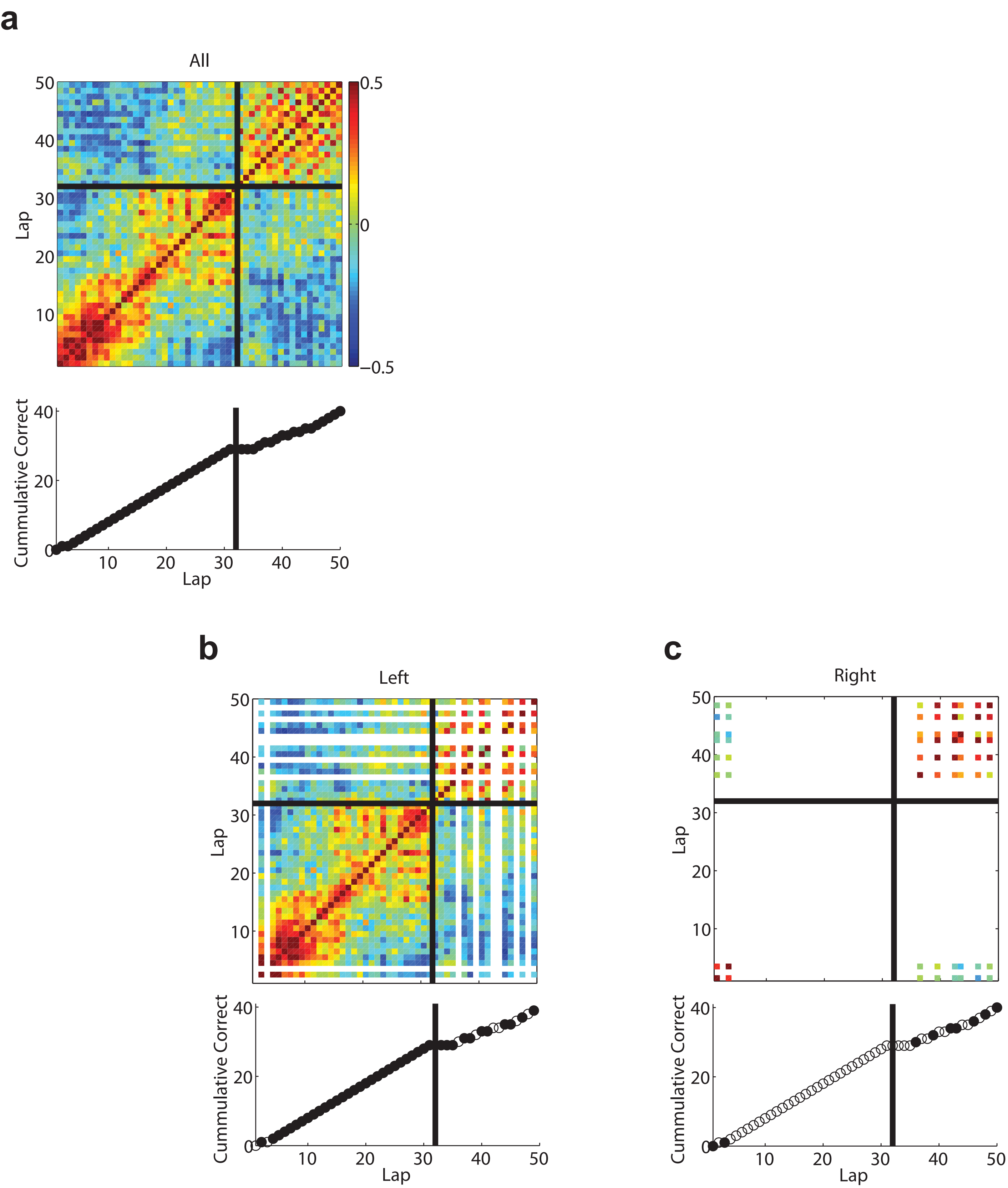

a
All
50
40
30
20
10
Lap
0.5
0
−0.5
Cummulative Correct
40
20
0
10
20
30
40
50
Lap
b
Left
c
Right

SUPPLEMENTARY FIGURE 6

Behavioral controls, MT-LRA task. a: Cross-correlation and behavior plots for a typical session, showing a sharp representational transition at the switch lap. This was a leftward to alternation reward contingency session. **b**: Cross-correlation and behavior plots for leftward only laps on that same session. Rightward laps were removed from the correlation and are shown with open circles in the behavior plot. Note that there is low correlation between leftward laps before the switch and leftward laps after the switch. **c**: Cross-correlation and behavior plots for rightward only laps on that same session. Here, leftward laps were removed from the correlation plot and are shown with open circles in the behavior plot. Note that rightward plots in the early laps were negatively correlated with the rightward plots after the transition.

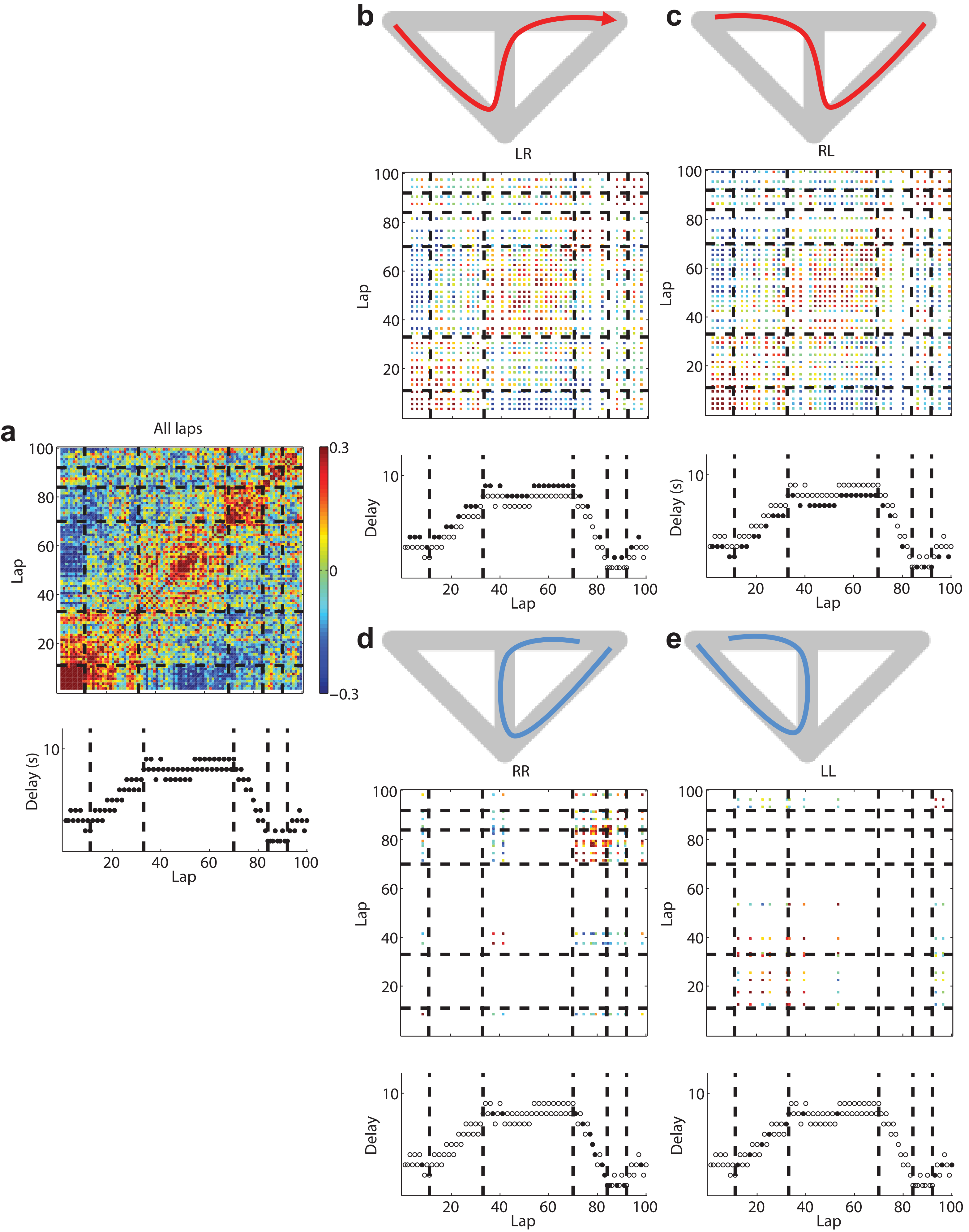

a
All laps
Lap
Delay (s)
Lap
b
LR
Lap
Delay
Lap
c
RL
Lap
Delay (s)
Lap
d
RR
Lap
Delay
Lap
e
LL
Lap
Delay
Lap

SUPPLEMENTARY FIGURE 7

Behavioral controls, DD task. a: Cross-correlation and behavior plots for a typical session, showing sharp representational transitions. This was a rare session in which rats showed both upward and downward titration.   We selected this session for this example because all four potential paths (LL, RR, LR, RL) occurred in all strategic phases on this session. Additionally, the delay on the DD was cue by tones (see Methods), and thus one might argue that the change in the delay length to the high-value reward in the DD task is a cue that could account for the representational changes seen in our ensemble. However, on this day, titration upward through the same delays as downward titration produced negatively correlated representations. **b-e**: Cross-correlation and behavior plots for each of the four lap types (**b**: left to right [LR], **c**: right to left [RL], **d**: right to right [RR], and **e**: left to left [LL]).
